# A Mycobacterial Perspective on Tuberculosis in West Africa: Significant Geographical Variation of *M*. *africanum* and Other *M*. *tuberculosis* Complex Lineages

**DOI:** 10.1371/journal.pntd.0004408

**Published:** 2016-03-10

**Authors:** Florian Gehre, Samrat Kumar, Lindsay Kendall, Mebrat Ejo, Oumie Secka, Boatema Ofori-Anyinam, Emmanuel Abatih, Martin Antonio, Dirk Berkvens, Bouke C. de Jong

**Affiliations:** 1 Mycobacterial Unit, Biomedical Sciences, Institute of Tropical Medicine, Antwerp, Belgium; 2 Vaccines and Immunity Theme, Medical Research Council (MRC) Unit, Fajara, The Gambia; 3 Biomedical Sciences, Institute of Tropical Medicine, Antwerp, Belgium; 4 Statistics and Bioinformatics Department, Medical Research Council (MRC) Unit, Fajara, The Gambia; 5 University of Gondar, Gondar, Ethiopia; 6 Division of Infectious Diseases, Department of Medicine, New York University (NYU), New York, New York, United States of America; European Bioinformatics Institute, UNITED KINGDOM

## Abstract

**Background:**

Phylogenetically distinct *Mycobacterium tuberculosis* lineages differ in their phenotypes and pathogenicity. Consequently, understanding mycobacterial population structures phylogeographically is essential for design, interpretation and generalizability of clinical trials. Comprehensive efforts are lacking to date to establish the West African mycobacterial population structure on a sub-continental scale, which has diagnostic implications and can inform the design of clinical TB trials.

**Methodology/Principal Findings:**

We collated novel and published genotyping (spoligotyping) data and classified spoligotypes into mycobacterial lineages/families using TBLineage and Spotclust, followed by phylogeographic analyses using statistics (logistic regression) and lineage axis plot analysis in GenGIS, in which a phylogenetic tree constructed in MIRU-VNTRplus was analysed. Combining spoligotyping data from 16 previously published studies with novel data from The Gambia, we obtained a total of 3580 isolates from 12 countries and identified 6 lineages comprising 32 families. By using stringent analytical tools we demonstrate for the first time a significant phylogeographic separation between western and eastern West Africa not only of the two *M*. *africanum* (West Africa 1 and 2) but also of several major *M*. *tuberculosis sensu stricto* families, such as LAM10 and Haarlem 3. Moreover, in a longitudinal logistic regression analysis for grouped data we showed that *M*. *africanum* West Africa 2 remains a persistent health concern.

**Conclusions/Significance:**

Because of the geographical divide of the mycobacterial populations in West Africa, individual research findings from one country cannot be generalized across the whole region. The unequal geographical family distribution should be considered in placement and design of future clinical trials in West Africa.

## Author Summary

Tuberculosis is caused by bacteria belonging to the *Mycobacterium tuberculosis* complex (MTBc), which consists of seven major, phylogenetically distinct lineages and their families. West Africa is the only region in the world where, besides the common *M*. *tuberculosis* lineages, the two *M*. *africanum* lineages are endemic. We demonstrate that the composition of the mycobacterial population in the western part of West Africa significantly differs from the one in the eastern part. This documented variation will impact on generalizability and interpretation of clinical trials outcomes. Therefore future trial designs need to consider the geographical diversity of underlying mycobacterial populations.

## Introduction

West Africa consists of 15 countries with 245 million inhabitants (S1A Fig), 13 of which belong to the world’s 42 countries with the lowest human development index [1]. Consequently, it faces great challenges in controlling infectious diseases, such as tuberculosis (TB). Clinical trials investigating the local health needs are much needed to understand and tackle the TB epidemic in West Africa.

The composition of the endemic mycobacterial population infecting human study subjects can have a major impact on TB clinical trial outcomes and should ideally be accounted for in the planning phase of any project [2]. Considering bacterial variation between study sites is also essential to estimate to what extent country-specific results can be generalised to the whole of West Africa.

The MTBc can be divided into six major lineages, comprised of the Indo-Oceanic (L1), East-Asian (L2), Central Asian (L3), Euro-American lineages (L4) and the two endemic African lineages *M*. *africanum* West Africa 1 (MAF1, L5) and *M*. *africanum* West Africa 2 (MAF2, L6) [3]. Although MAF1 seems to be disappearing in some countries, the longitudinal development of MAF2 is not known. Each of these phylogenetically distinct lineages can be further differentiated into mycobacterial families, such as, amongst others, the Latin-American-Mediterranean (LAM) or Haarlem families within the Euro-American lineage [3].

Interestingly and for reasons not understood, West Africa is the only region in the world in which all of the six major human lineages are present. This exceptional diversity necessitates future West African trials to be adjusted for this unique bacterial variability—even more than trials in other parts in the world. Therefore the scope of the present publication was to describe the geographical distribution and spatial variations of mycobacterial families across the region.

## Methods

### Search strategy and spoligotype analysis

We searched Pubmed using terms “spoligotype”, “spoligotyping” with respective country names. Studies on pulmonary TB up to December 2014 were included, in which spoligotypes on all isolates were available. Individual spoligotypes designated as mixed infections were excluded. In case several publications analysed the same dataset, the most comprehensive collection was selected. *M*. *bovis* studies, conducted in high risk populations (abattoir staff) were excluded. To assign mycobacterial families to isolates, and to ensure comparability between different datasets, we re-analysed extracted spoligotype information using a standardized approach. Isolates were classified into families using the online platform “Spotclust” at the default settings. For *M*. *africanum* isolates, Spotclust identifies, but does not distinguish between MAF1 and 2. Therefore “TBLineage” was further applied to *M*. *africanum* isolates previously identified by Spotclust [4]. Both Spotclust and TB Lineage are mathematical algorithms that were shown to reliably identify mycobacterial lineages and families based on respective signature spoligotype patterns. A detailed description of the algorithms and their performance is described elsewhere [4,5]. The lineage/family distribution per country/study site was plotted as chloropleth maps generated using QGIS 2.0.1 (http://qgis.osgeo.org).

### Statistical analysis of geographical genotype distribution

To investigate geographical differences in mycobacterial families across West Africa we split West Africa into a Western and an Eastern region. Western countries include Gambia, Guinea-Bissau, Guinea, Sierra Leone, Ivory Coast, Mali, Senegal, while Eastern countries include Benin, Burkina Faso, Ghana, Niger and Nigeria (S1 Fig). With region as response variable, the proportion of each family was tested univariately using logistic regression, with country fitted as a cluster to account for multiple studies per site.

Families found in one region and not in the other cannot be modelled mathematically because the maximum likelihood for these families does not exist. We defined families with complete separation between regions as ‘perfect predictors’.

A two-sided p-value <0.05 was considered statistically significant and a two-sided p-value ≥0.05 & <0.10 was considered of borderline significance. No adjustment was made for multiple testing. All analyses were performed using Stata v12.1 (StataCorp. 2011. *Stata Statistical Software*: *Release 12*. College Station, TX: StataCorp LP.).

### GenGIS analysis

Phylogeographic analysis using linear axis analysis in GenGISvs2.2.2 was conducted [6]. The default GenGIS Africa map was used. A UPGMA phylogenetic tree was constructed from spoligotyping data (S2 Fig) using the publicly available MIRU-VNTRplus software [7] and uploaded into GenGIS allowing for the re-ordering of leaf nodes. A Linear axis plot (10.000 permutations) was run at significance level p = 0.001.

### Longitudinal analysis of lineages in The Gambia

Gambian isolates, collected within a TB Case Contact cohort, in which all cases of the Greater Banjul area are recruited [8] were spoligotyped. Genotyping was approved by the Gambian Government/MRC joint ethics committee. Longitudinal lineage data was modelled using logistic regression for grouped data. The outcome was the number of a particular lineage out of the total number of samples taken in each year. Both lineage and year were fitted as explanatory variables and interactions between the two explored. The multicollinearity between the lineages was avoided by excluding one lineage and fitting the model on the remaining.

## Results

### The *M*. *tuberculosis* complex in West Africa

**Fig 1:**
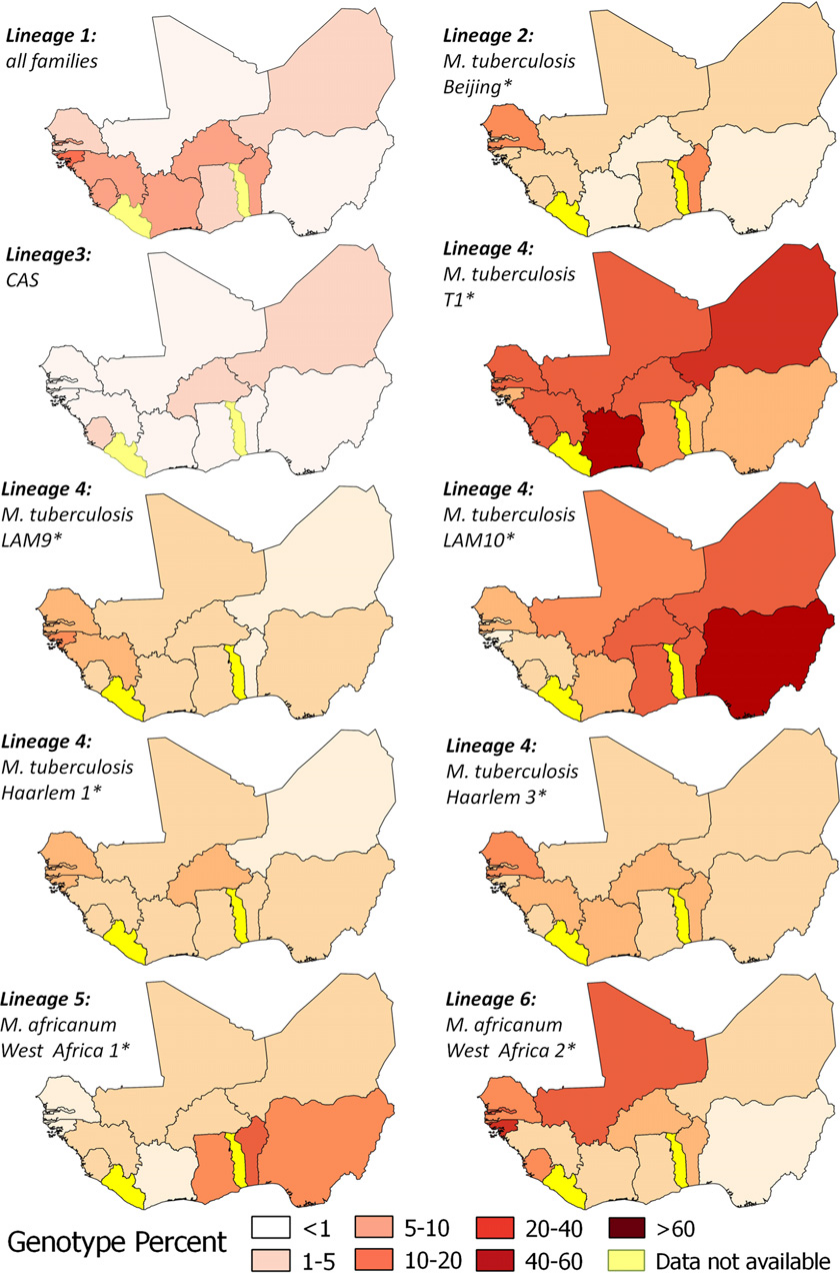
Geospatial distribution of major mycobacterial families within each lineage in West Africa. For each of the six present lineages, the major families are mapped. The eight overall major families highlighted with asterisks (*) cause 84% of all pulmonary TB in the region and comprise *M*. *africanum* West Africa 1 (MAF1), *M*. *africanum* West Africa 2 (MAF2), LAM9, LAM10, Haarlem 1, Haarlem 3 and Beijing families.

Of 20 original research articles, four were excluded (based on above criteria), with the remaining 16 covering 12 of 15 West African countries. In total we collected, extracted and (re)analysed spoligotype information of 3580 isolates, belonging to six major human lineages, of which the Euro-American lineage (L4), together with *M*. *africanum* lineages (L5 and 6) were the main causes of pulmonary TB (Table 1). Thirty-two different mycobacterial families were identified, but 84% of all patients are infected by only eight major families (Fig 1).

**Table 1:** Geospatial distribution (absolute numbers) of 3580 spoligotypes belonging to 6 identified West African mycobacterial lineages (L1-L6) and 32 families, which were East African Indian (EAI) families EAI 1, EAI 2, EAI 4, EAI 5; Family 33, Family 34, Family 35, Family 36; Beijing family; Central Asian (CAS) family; Haarlem (H) families H1, H2, H3; H37Rv-like family; Latin-American-Mediterranean (LAM) families LAM 1, LAM 2, LAM 3, LAM 7, LAM 8, LAM 9, LAM 10; S family; T families T1, T2, T3, T4; X families X1, X2, X3; *M*. *africanum* West Africa 1 and 2; *M*. *bovis*.

				Mycobacterial Lineages																															
				L1							L2	L3	L4																				L5	L6	Animal strains
				Indo-Oceanic							East Asian	Central Asian	Euro American																				*M*. *africanum* West Africa		
		Mycobacterial Family		East African Indian (EAI)				Family			Beijing	CAS	Haarlem			H37Rv-like	Latin-American-Mediterranean (LAM)							S	Family	T families				X families					*M*. *bovis*
Site ID	Ref.	Study year	No. Study isolates	1	2	4	5	33	34	35			H1	H2	H3		1	2	3	7	8	9	10		36	T1	T2	T3	T4	X1	X2	X3	1	2	
**Benin**	[9]	2005–2006	192	0	0	0	4	6	1	0	20	0	2	0	13	1	0	0	0	0	2	0	56	0	1	10	0	0	1	1	0	2	60	12	0
**Burkina Faso**	[10]	2001	80	0	0	0	0	4	0	0	1	5	8	0	3	0	0	0	0	0	0	1	19	0	0	17	0	0	0	1	0	4	2	14	1
	[11]	2010	119	2	0	0	1	5	0	0	0	2	8	0	10	1	0	0	0	1	1	1	43	3	0	35	0	0	0	0	0	4	0	2	0
**Cape Verde**	No data available		-	-	-	-	-	-	-	-	-	-	-	-	-	-	-	-	-	-	-	-	-	-	-	-	-	-	-	-	-	-	-	-	-
**The Gambia**	[12]	2002–2009	884	3	0	2	12	12	11	0	26	2	63	6	67	5	38	15	5	14	11	71	34	1	1	113	6	31	1	0	1	7	2	324	0
	This study	2012	280	0	0	0	2	5	3	0	3	0	24	0	33	1	14	5	2	0	7	32	6	1	1	37	1	7	2	1	1	1	3	88	0
**Ghana**	[13]	2007–2009	162	3	0	0	7	0	0	0	4	1	8	0	8	0	0	0	2	0	1	2	58	2	1	28	0	0	0	1	0	9	23	4	0
**Guinea-Bissau**	[14]	1989–2008	414	6	0	1	27	7	6	0	7	0	22	0	16	0	11	2	1	0	2	59	2	1	1	40	8	0	0	3	0	2	1	189	0
**Guinea**	[15]	2005–2010	120	2	0	0	0	0	10	0	6	0	5	0	12	1	4	0	4	0	2	8	6	3	0	34	9	0	3	0	2	1	3	5	0
**Ivory Coast**	[16]	2008–2009	194	0	0	0	2	0	0	0	3	1	5	0	5	1	0	0	0	0	0	0	14	2	1	151	2	0	1	0	0	2	0	4	0
	[17]	1994–1995	20	0	0	0	0	0	0	0	0	0	0	0	2	1	0	0	0	0	0	2	1	0	0	13	0	0	0	0	0	1	0	0	0
**Liberia**	No data available		-	-	-	-	-	-	-	-	-	-	-	-	-	-	-	-	-	-	-	-	-	-	-	-	-	-	-	-	-	-	-	-	-
**Mali**	[18]	2006–2010	126	0	1	0	3	0	0	0	2	2	2	0	4	1	1	0	0	1	1	3	15	1	0	47	2	0	1	1	0	1	2	34	1
**Niger**	[15]	2008–2012	87	0	0	0	0	0	0	0	1	0	0	0	3	0	0	0	0	0	0	0	30	0	0	49	0	0	0	0	0	1	1	2	0
**Nigeria**	[19]	2006–2008	111	0	0	0	2	4	0	0	0	0	6	0	4	0	0	0	0	0	4	2	79	0	0	2	2	0	0	0	0	5	1	0	0
	[20]	2006	60	5	0	0	0	1	0	0	0	0	2	0	2	0	0	0	0	0	2	1	35	0	0	6	0	0	0	1	0	0	2	0	3
	[21]	2009–2010	405^1^	6	0	1	0	1	0	2	1	0	13	0	21	0	0	0	0	0	4	3	256	0	0	32	6	1	0	0	0	8	46	0	4
	[22]	2008–2009	81	0	0	0	1	1	0	1	0	0	2	0	3	0	0	0	0	0	2	0	41	0	0	2	1	0	0	0	0	1	26	0	0
**Senegal**	[15]	1997–2011	79	0	0	0	0	1	0	0	11	3	3	2	9	1	0	0	0	2	2	7	5	1	0	22	0	0	0	0	1	0	0	9	0
	[17]	1994–1995	69	0	0	0	2	6	0	0	8	1	7	0	6	2	2	2	0	0	1	6	5	0	0	14	0	0	0	0	0	0	0	7	0
**Sierra Leone**	[23]	2003–2004	97	0	0	0	4	0	3	1	4	0	2	0	3	0	3	0	3	0	2	4	4	5	0	27	8	0	1	1	1	0	4	17	0
**Togo**	No data available		-	-	-	-	-	-	-	-	-	-	-	-	-	-	-	-	-	-	-	-	-	-	-	-	-	-	-	-	-	-	-	-	-
**Total No of Isolates**			3580	27	1	7	67	53	34	4	97	17	182	8	224	15	73	24	17	18	44	202	709	20	6	679	45	39	10	10	6	49	175	712	8

^1^excluding designated mixed infections

Common to most of the countries is the “ill-defined” T1 family. We also confirmed the previously described geographical distribution of two *M*. *africanum* lineages [24]. While MAF1 (L5) has the highest presence in Nigeria/Benin, MAF2 (L6) is mainly found in Gambia/Guinea-Bissau. Besides MAF2 as a major cause for TB, a variety of Euro-American families (Haarlem 1 and 3, LAM9, amongst others) are prevalent in western West Africa. This is in sharp contrast to eastern West Africa where, besides MAF1, the great majority of TB infections is attributable to only one other dominant family LAM10. A recently introduced family into West Africa is the Beijing family which lead to an outbreak in Cotonou, Benin [25]. The only other place with comparably high numbers of Beijing isolates is Dakar in Senegal. Both cities, Dakar and Cotonou have major international ports.

### Geographical distribution of mycobacterial families in West Africa

To evaluate whether identified families are geographically equal, we divided West Africa into a Western and Eastern region (S1B Fig). Univariate logistic regression analysis showed that the proportion of mycobacterial families can serve as predictors for the two regions. 13 out of 32 families were associated with one of the two regions (see S1 Table). Amongst these were four of the eight major families: LAM10 (perfect predictor at proportion ≥0.12) and MAF1 (p = 0.08) as predictors for the East and Haarlem 3 (p = 0.07) and MAF2 (p = 0.09) for the West.

**Fig 2:**
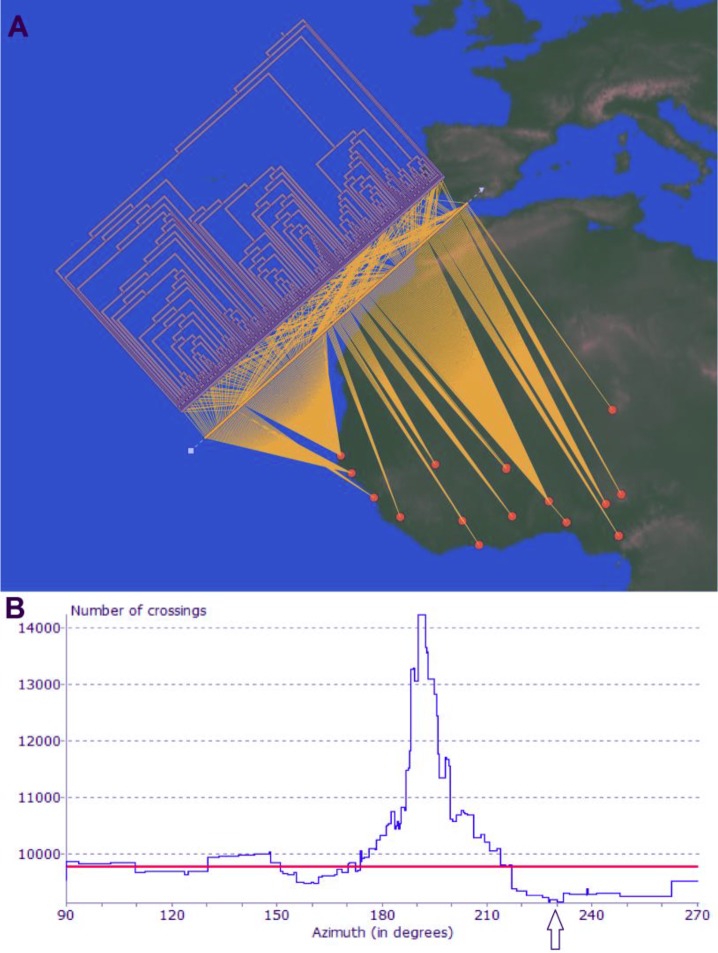
Phylogeographic analysis of major geographically restricted families using GenGIS. **A.)** Phylogeography, in which an UPGMA tree that includes MAF1, MAF2, Haarlem 3 and LAM10 spoligotypes, is superimposed onto the geographical distribution map. Each spoligotype in the tree is linked to its actual location on the map and the crossing-overs of connecting lines can be counted. In case of no geographical separation crossings of the connecting lines would occur at random. If less crossings are observed than expected by chance geographical separation occurs, as for instance, at a geographic tree axis angle of 228.1° of these major four families. **B)** Linear axis plot scanning for axis angles of the superimposed phylogenetic tree onto the map, in which geographic separation occurs. The red line indicates the minimum number of crossings that would have been expected by chance at significance level p = 0.001. Every orientation of the tree onto the map that results in less crossings than expected by chance (9759.5) lies below the line and indicates significant geographical separation and can be observed between 110°-131°, 151°-170° and 217°- 270°. The most extreme geographical separation with the least crossings (9144) occurs at an angle of 228.1° (arrow) and is plotted in 2A.

To verify the geographic separation of these four major families, which cause 51% of all TB, we carried out an independent phylogeographic analysis using GenGIS software (Fig 2). We constructed an UPGMA tree based on 279 unique Haarlem 3, MAF1/2 and LAM10 spoligotypes, which was superimposed onto geographic locations and mycobacterial family distributions of the study sites (Fig 2A). In case of geographical separation, one expects significantly less crossings between the phylogenetic tree and the spoligotype distribution in the study sites than by mere chance. A linear axis analysis (p<0.001, 10.000 permutations) identified several orientations of the tree’s geographical axis that resulted in less than the 9759.5 crossings expected by chance. Fig 2B demonstrates that geographical separation occurs at various geographical axis angles, with the least crossings (9144) at 228.1° (Fig 2A). Although spoligotyping might have led to minor misclassifications of MAF1/MAF2 isolates in our phylogenetic analyses (S2 Fig), we expect such misclassification to result in an unbiased underestimation of the observed geographical separation.

### Longitudinal development of lineages in The Gambia

**Fig 3:**
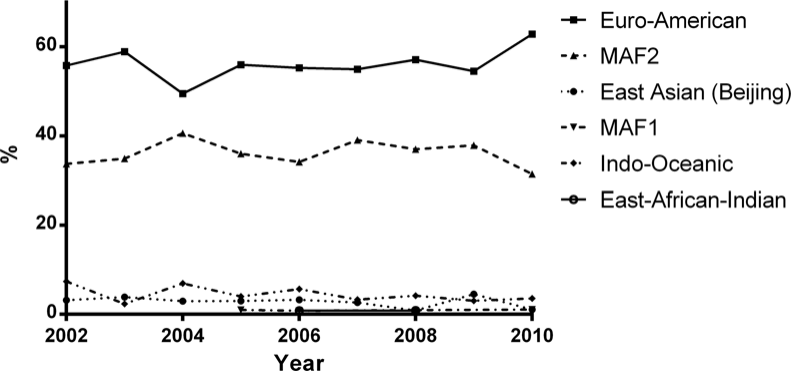
Longitudinal development of the *M*. *tuberculosis* complex in The Gambia between 2002–2010. 1164 smear-positive pulmonary TB isolates were spoligotyped and assigned to lineages. We analyzed the longitudinal development of the six prevalent lineages over time, using logistic regression modelling for grouped data. As no lineage/time interaction or time as main effect was detected, the average prevalence and 95% confidence intervals for each lineage were estimated as follows: Euro-American: 57.2% (54.4%-60.0%); *M*. *africanum* West Africa 2 (MAF2): 35.4% (32.7%-38.2%); Indo-Oceanic: 4.3% (3.3%-5.6%); East Asian (Beijing): 2.5% (1.7%-3.6%); *M*. *africanum* West Africa 1 (MAF1): 1.0% (0.4%-2.4%); East African Indian 0.8% (0.2%-3.2%).

1164 consecutive TB patients were recruited between 2002–2010. Logistic regression modelling revealed both a non-significant lineage by time interaction (p = 0.38) and a non-significant time main effect (p = 0.80). Our analysis therefore indicated that the proportions of lineages are stable over time. The overall lineage percentages, in order of magnitude, are Euro-American 57.2% (95%CI 54.4%-60.0%), MAF2 35.4% (95%CI 32.7%-38.2%), Indo-Oceanic 4.3% (95%CI 3.3%-5.6%), East Asian (Beijing) 2.5% (95%CI 1.7%-3.6%), MAF1 1.0% (95%CI 0.4%-2.4%) and East African Indian 0.8% (95%CI 0.2%-3.2%) (Fig 3).

## Discussion

We confirmed that modern Euro-American strains are the predominant lineage followed by the two *M*. *africanum* lineages. Although the polyphyletic T1 family [26] is rather equally distributed across the whole region, we find geographical variations of other families. While western West Africa shows a high genetic diversity from a multitude of mycobacterial families, the MTBc of Eastern West Africa is mainly composed of two dominant families (LAM10 and MAF1). Although other West and Central African countries observed a replacement of MAF1 and MAF2 with modern strains [10,11,14,27], our longitudinal analysis from The Gambia did not confirm these findings and MAF2 remains an important cause of TB in the country. The exact mechanism of how MAF2 can maintain a stable prevalence of 35% over the last decade within The Gambia (despite a slower progression to disease when compared to *M*. *tuberculosis* [28]) is not fully understood.

Besides the known geographical divide of the two *M*. *africanum* lineages, we find for the first time geographical separation of major Euro-American families in West Africa. Due to this spatial variation previous research findings observed in one West African country/region are hardly generalizable to the sub-region. In addition, the unequal distribution has important implications for design of future trials. For instance, western West African countries with their high genetic diversity are appropriate settings for research that aims to test whether novel diagnostics or vaccine candidates work equally well against different MTBc families. In contrast, research on host genetics, benefitting from low diversity, would yield more robust results when conducted in eastern West Africa with predominant LAM10 and MAF1 families. To investigate the spreading of novel TB families, one can follow up on the geographical expansion of LAM10 or on recently introduced Beijing strains into Benin or Senegal. As first studies confirmed that the “ill-defined”T1 is not a monophyletic clade [26], further research using more robust phylogenetic markers could focus on understanding the endemic MTBc composition T1-endemic countries.

The presented phylogeography also has limitations: first, we combined genotypic information, independent from respective collection strategies ranging from convenience to systematic sampling. Therefore data presented are a cross-sectional compilation of genotyping information between 1986–2012. Also, individual patient’s treatment history, whether they presented as new or retreatment cases, was not systematically collected and has not been accounted for. In order to avoid over-interpretation of results, we agree that comparing differing sampling strategies is challenging, and we therefore limited our discussion to proportions of families with larger isolate numbers. Lastly, the families themselves consist of a multitude of strains characterised by specific spoligotypes (shared international types, SITs) and we did not study whether the local expansion of a family was driven by one or several individual proliferating SIT within the family.

Spoligotyping can be successfully used to assign the majority of mycobacterial isolates to one of the major mycobacterial lineages and their families [4]. We appreciate that classification of mycobacteria in West Africa would ideally be based on whole genome sequencing (WGS) data, however, limited bioinformatics capacity combined with financial and infrastructural constraints did not allow high-throughput sequencing in most resource-limited West African countries to date.

By summarizing available and novel data, we showed significant geographical variation of the MTBc, which will impact on the overall outcome of clinical trials in any specific region. With the generated data researchers can consider the demonstrated spatial variation in the planning stage of respective future clinical TB trials.

## Supporting Information

S1 Fig **A:** Map of West Africa. West Africa consists of 15 countries, for 13 of which data was available for the present study; **B:** For the analysis of geographical separation of families we divided West Africa into a Western part (“blue”—Senegal, The Gambia, Guinea-Bissau, Guinea, Sierra Leone, Ivory Coast, Mali) and an Eastern part (“red”—Burkina Faso, Ghana, Benin, Niger and Nigeria).
(TIF)

S2 Fig UPGMA tree of 279 different Haarlem 3, *M*. *africanum* West Africa 1 & 2 and LAM10 spoligotypes used for GenGIS analysis. Leaves of the tree are labelled with the respective mycobacterial families. Due to the limitations of spoligotyping and misclassification of strains, one clade contains a mixture of MAF1 and MAF2 isolates. For the use in GenGIS we allowed for re-ordering of the leaf nodes, i.e. flipping of sub-trees around a leaf node.
(PNG)

S1 Table Univariate logistic regression analysis demonstrating that presence of genotypes can serve as significant predictors for Western (Gambia, Guinea-Bissau, Guinea, Sierra Leone, Ivory Coast, Mali, Senegal) and Eastern (Benin, Burkina Faso, Ghana, Niger, Nigeria) West Africa. (DOCX)
