# Supplementary figures and images for: A Mycobacterial Perspective on Tuberculosis in West Africa: Significant Geographical Variation of M. africanum and Other M. tuberculosis Complex Lineages
Source: PLoS Negl Trop Dis. 2016 Mar 10;10(3):e0004408. doi: 10.1371/journal.pntd.0004408 (PMC4786107; doi:10.1371/journal.pntd.0004408)

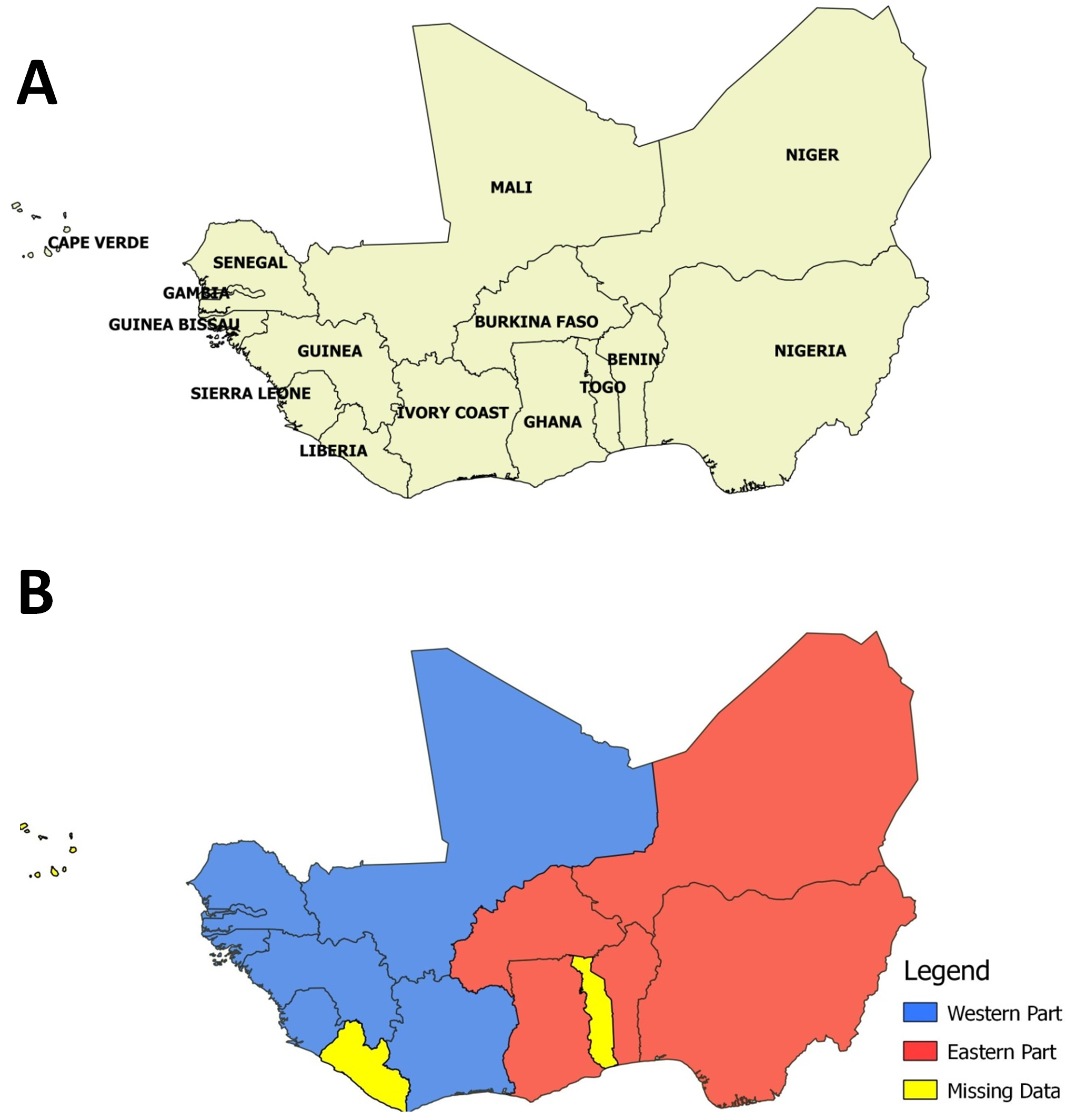

Supplement: S1 Fig — A: Map of West Africa. West Africa consists of 15 countries, for 13 of which data was available for the present study; B: For the analysis of geographical separation of families we divided West Africa into a Western part (“blue”—Senegal, The Gambia, Guinea-Bissau, Guinea, Sierra Leone, Ivory Coast, Mali) and an Eastern part (“red”—Burkina Faso, Ghana, Benin, Niger and Nigeria). (TIF) [file pntd.0004408.s001.tif]

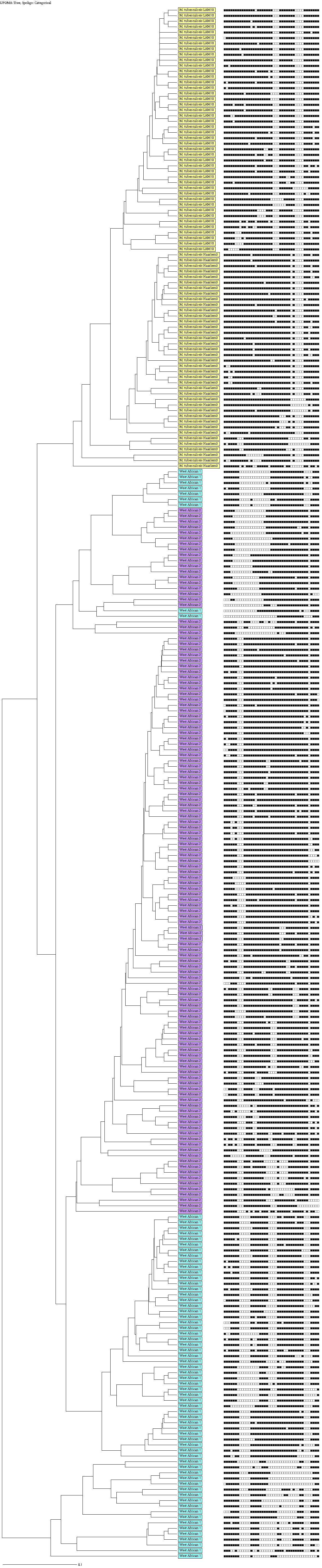

Supplement: S2 Fig — Leaves of the tree are labelled with the respective mycobacterial families. Due to the limitations of spoligotyping and misclassification of strains, one clade contains a mixture of MAF1 and MAF2 isolates. For the use in GenGIS we allowed for re-ordering of the leaf nodes, i.e. flipping of sub-trees around a leaf node. (PNG) [file pntd.0004408.s002.png]
