# Supplementary material for: A Mycobacterial Perspective on Tuberculosis in West Africa: Significant Geographical Variation of M. africanum and Other M. tuberculosis Complex Lineages
Source: PLoS Negl Trop Dis. 2016 Mar 10;10(3):e0004408. doi: 10.1371/journal.pntd.0004408 (PMC4786107; doi:10.1371/journal.pntd.0004408)
Supplement: S1 Table — (DOCX) [file pntd.0004408.s003.docx]

Supplementary Table S1: Univariate logistic regression analysis demonstrating that presence of genotypes can serve as significant predictors for Western (Gambia, Guinea-Bissau, Guinea, Sierra Leone, Ivory Coast, Mali, Senegal) and Eastern (Benin, Burkina Faso, Ghana, Niger, Nigeria) West Africa

| **Mycobacterial family** | **Perfect predictor** | **Significant, α=0.05** | **Significant, α=0.10** | **Not significant** | **Predicted region** |
| --- | --- | --- | --- | --- | --- |
| Beijing |  |  |  | 0.35 | - |
| *M. bovis* |  |  |  | 0.16 | - |
| H37Rvlike |  |  | 0.08 |  | West |
| S |  |  |  | 0.25 | - |
| CAS |  |  |  | 0.83 | - |
| EAI1 |  |  |  | 0.20 | - |
| EAI2 | x |  |  |  | East |
| EAI4 |  |  |  | 0.56 | - |
| EAI5 |  |  |  | 0.35 | - |
| Family 33 |  |  |  | 0.64 | - |
| Family 34 |  |  | 0.06 |  | West |
| Family 35 |  |  |  | 0.61 | - |
| Family 36 |  |  |  | 0.97 | - |
| Haarlem 1 |  |  |  | 0.81 | - |
| Haarlem 2 | x |  |  |  | West |
| Haarlem 3 |  |  | 0.07 |  | West |
| LAM1 | x |  |  |  | West |
| LAM2 | x |  |  |  | West |
| LAM3 |  |  |  | 0.24 | - |
| LAM7 |  |  |  | 0.12 | - |
| LAM8 |  |  |  | 0.82 | - |
| LAM9 |  |  |  | 0.13 | - |
| LAM10^1^ | x |  |  |  | East |
| MAF1 |  |  | 0.08 |  | East |
| MAF2 |  |  | 0.09 |  | West |
| T1 |  |  |  | 0.26 | - |
| T2 |  |  |  | 0.21 | - |
| T3 |  | 0.006 |  |  | West |
| T4 |  |  | 0.09 |  | West |
| X1 |  |  |  | 0.37 | - |
| X2 | x |  |  |  | West |
| X3 |  |  |  | 0.13 | - |

# ^1^at proportion ≥0.12
